# Validation and Refinement of Scores to Predict Stroke Risk: Prospective Cohort Study

**DOI:** 10.2196/72497

**Published:** 2025-08-21

**Authors:** Hua Meng, Zhuo Liu, Dongfeng Pan, Xinya Su, Wenwen Lu, Xingtian Wang, Yuhui Geng, Xiaojuan Ma, Peifeng Liang

**Affiliations:** 1 Hubei Provincial Clinical Research Center for Alzheimer’s Disease, Tianyou Hospital, School of Medicine, Wuhan University of Science and Technology, Wuhan, China; 2 Brain Science and Advanced Technology Institute, Wuhan University of Science and Technology, Wuhan, China; 3 School of Public Health, Ningxia Medical University, Yinchuan, China; 4 Department of Emergency Medicine, People's Hospital of Ningxia Hui Autonomous Region, Ningxia Medical University, Yinchuan, China; 5 Futian Center for Chronic Disease Control, Shenzhen, China; 6 Medical Record Statistics Department, General hospital of Ningxia Medical University, Yinchuan, China; 7 Department of Medical Affair, People's Hospital of Ningxia Hui Autonomous Region, Ningxia Medical Univeristy, 301 Zhengyuan North Street, Yinchuan, 750002, China, 86 13895085519

**Keywords:** stroke, Rothman-Keller model, logistics model, risk prediction, “8+2” stroke risk score

## Abstract

**Background:**

In China, the “8+2” stroke risk score has been widely used to identify individuals at high risk of stroke, despite insufficient evidence confirming its predictive ability for stroke events.

**Objective:**

We aimed to validate the risk score’s ability to predict the risk of stroke within a 10-year timeframe in community cohort populations and to optimize the scoring method to improve its predictive accuracy.

**Methods:**

By reviewing previous literature to obtain the parameters for constructing the logistic regression model and the Rothman-Keller model, the risk threshold points of the models were determined using a sample of 100,000 participants. For this population-based cohort study, 22,259 community residents were recruited in 2013 from one urban and rural monitoring site in Ningxia, China. The occurrence of stroke was established by a combination of self-reporting and review of hospitalization electronic records (the *International Statistical Classification of Diseases and Related Health Problems 10th Revision*: I60-63). A logistic regression model and a Rothman-Keller model were used to refine the 8-factor stroke risk score to predict the 10-year stroke risk. The performance of the model was assessed by the area under the receiver operating characteristic curve and net reclassification improvement.

**Results:**

The threshold points for low and medium risk in the logistic regression model and the Rothman-Keller model are risk scores of 0.062 and 0.002, respectively. The threshold points for medium and high risk are risk scores of 0.165 and 0.005, respectively. A total of 11,692 community residents aged 40 years or older who met the inclusion criteria completed the 10-year follow-up. According to the “8+2” stroke risk score, the stroke incidence in the low-risk (n=8908), medium-risk (n=1074), and high-risk groups (n=1710) was 4.5%, 14.7%, and 12.3%, respectively. The logistic regression model and the Rothman-Keller model demonstrated significant differences in area under the receiver operating characteristic curve values when compared to the “8+2” stroke risk score (Z=2.60, *P*=.001; Z=3.47, *P*=.009, respectively). However, no significant difference was observed between the logistic regression model and the Rothman-Keller model (Z=0.688, *P*=.49). Relative to the risk score, the absolute net reclassification improvement of the Rothman-Keller model was 0.051 (*P*=.01) and of the logistic regression model was 0.010 (*P*=.62).

**Conclusions:**

Our study confirmed that the “8+2” stroke risk score does not effectively predict stroke events. But the Rothman-Keller model may enhance the ability to identify individuals at high risk for stroke. Future research should incorporate more specific biomarkers and multimodal imaging features to develop more accurate risk prediction models.

## Introduction

The Global Burden of Disease (GBD) 2021 study found that the number of new cases of stroke increased by 70.2% from 1990 to 2021 [1], highlighting a relatively insufficient emphasis on prevention, particularly in low-income countries. The number of patients with stroke in China is currently the highest in the world. Stroke is the leading cause of death and disability among adults in the country [2]. China has the highest risk of stroke globally, with an overall lifetime risk of 39.9% [3]. Consequently, China has implemented a series of measures to address the increasing burden of stroke, advocating for an integrated approach to stroke prevention, treatment, management, and rehabilitation [4].

The National Ministry of Health of China launched a significant national project called the “China National Stroke Screening Survey (CNSSS)” in 2009 to tackle the challenge posed by stroke. The China Stroke Prevention Project Committee (CSPPC) was established in April 2011. Volunteers aged 40 years and older were recruited through structured face-to-face questionnaires, and the “8+2” risk scorecard is used to screen participants and to identify high-risk groups [5]. The “8” refers to 8 risk factors (hypertension, heart disease, smoking, dyslipidemia, diabetes, physical inactivity, overweight, and family history of stroke [FHS]), and the “2” refers to transient ischemic attacks (TIAs) and previous strokes. According to the judging criteria, respondents are categorized into low-risk, medium-risk, and high-risk groups. In 2020, a total of 268,000 individuals in the high-risk group for stroke were identified across more than 240 project areas across the country [6].

Risk assessment is an effective tool for identifying prevention priorities [7]. The Framingham Stroke Risk Profile is recognized as one of the earliest and most widely used simple stroke risk assessment tools. However, validation studies in domestic populations have found that it tends to overestimate the actual stroke incidence to some extent [8]. Since its publication, the pooled cohort risk assessment equations have also been controversial, as some external validation studies suggest that this risk assessment model may overestimate the risk of atherosclerotic cardiovascular disease [9]. A study compared the performance of the Framingham cardiovascular risk equation, the pooled cohort equations, and the China-Population Attributable Risk equations in predicting the 5-year risk of atherosclerotic cardiovascular disease, including ischemic stroke. In the Uyghur and Kazakh populations, all 3 risk assessment equations consistently underestimated the risk [10]. Furthermore, although the “8+2” risk score tool has been widely used, its predictive ability remains unclear.

Therefore, we aimed to validate the “8+2” stroke risk score for predicting the 10-year risk of stroke in community cohort populations, and to optimize the scoring method to improve predictive accuracy.

## Methods

### Data Source

This study was a cohort study. This study followed the Transparent reporting of multivariable prediction model for Individual Prognosis or Diagnosis (TRIPOD) guidelines.

The cohort was part of the China Stroke High-risk Population Screening and Intervention Program (CSHPSIP), an ongoing nationwide population-based program [5,11]. The study participants were recruited from the screening site in the Jinfeng District of Yinchuan City, Ningxia Hui Autonomous Region. A total of 22,259 community residents were enrolled in 2013 from one urban and rural monitoring site, and the outcome ascertainment was completed in 2023. The inclusion criteria for the screening participants are individuals who are aged 40 or older, permanent residents (those who have lived in the area for 6 months or more), and those who voluntarily participate by signing an informed consent form [12]. Patients younger than 40 years, those with a history of stroke or TIA, or individuals recruited after 2014 were excluded from the study cohort. The data quality control process is detailed in the Multimedia Appendix 1.

### Risk Factors Measurement

Based on the “Stroke Screening and Prevention Technical Specifications” promulgated by the National Health and Family Planning Commission’s Stroke Screening and Prevention Engineering Committee, the following risk factors were assessed: hypertension, heart disease, smoking, dyslipidemia, diabetes, physical inactivity, overweight, and FHS. The detailed criteria for each risk factor are shown in Table S1 in Multimedia Appendix 1 which is also available on the China Stroke and Cardiovascular Disease website.

The criteria for classifying individuals into high-, medium-, and low-risk stroke groups were as follows: the high-risk group was defined as having 3 or more risk factors; the medium-risk group was characterized by 3 or less risk factors along with a history of chronic diseases (such as hypertension, diabetes, and heart disease); and the low-risk group was defined as having 3 or less risk factors without any history of chronic diseases (Table S1 in Multimedia Appendix 1) [13]. For high-risk individuals, follow-up visits are conducted by primary health care institutions at 6 months and 12 months after the initial assessment. For moderate-risk populations, primary health care institutions conduct a single follow-up visit at 12 months to evaluate and address their associated risk factors.

### Outcome

We recorded stroke as an endpoint event by searching electronic hospitalization records in Ningxia in June 2023. Stroke was identified using the diagnostic code I60-63 from the *International Statistical Classification of Diseases and Related Health Problems 10th Revision* (*ICD-10*).

### Logistic Regression Model

Logistic regression was used to determine the odds ratios (ORs) of every risk factor for incident stroke [14]. The basic equation for regression with multiple independent variables is:


$$Y=\ln\left(\frac{P}{1-P}\right)=\alpha +\beta_{1}X_{1}++\beta_{2}X_{2}+\cdots +\beta_{i}X_{i}$$

*Y* is the estimated continuous outcome; *α* is the intercept. This is considered a constant value; *β* is the beta coefficients; and *X_i_* is each risk factor.

### Rothman-Keller Model

The Rothman-Keller model, initially developed by Kenneth J. Rothman and David B. Keller in the early 1970s, was designed to assess the combined impact of tobacco and alcohol consumption on the risk of oral and pharyngeal cancers [15]. This model provides a statistical framework that enables researchers to quantify both the independent and joint contributions of various risk factors to disease risk. It has been adapted and applied to a wide range of health conditions and diseases, including early-onset colorectal cancer and mild cognitive impairment [16,17]. Its flexibility in considering both additive and multiplicative effects of risk factors makes it a valuable tool for public health research and individual risk prediction.

The Rothman-Keller model uses the binomial distribution function method for risk classification. It calculates the benchmark proportion of incidence and risk scores based on the population exposure rate and OR of each risk factor. In addition, it estimates an individual’s relative risk of developing a disease by calculating their combined risk scores. The parameters of the Rothman-Keller model are calculated as follows:

1. Baseline morbidity ratio (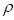
）：$\rho =\frac{1}{\sum_{i=1}^{n}OR_{i}\times P_{i}}=1-PAR\%$
  *P_i_:* the exposure rate of individuals exposed to a risk factor in the whole population; *OR_i_:* the odds ratios of exposure to a risk factor; and *PAR*%: population attributable risk percentage.
2. Risk score (）*：*$S=\rho \times \text{O}{\text{R}}_{i}$
3. Total risk score (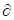
）：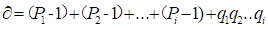  *Pi:* risk factor scores for S≥1, *q_i_*: risk factor scores for S<1.
4. Individual risk prediction score: Individual risk of stroke=the incidence of stroke × 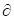. This expected risk of stroke is a relative value because it is measured against the overall incidence rate in the population. It can help us understand whether the individual’s likelihood of developing a stroke is higher or lower than the average level of the population.

The population exposure rate of every risk factor was derived from the literature [18]. The OR of exposure to a risk factor was sourced from the logistic regression model. Data for 100,000 participants were randomly generated by the binomial distribution functions of risk factors collected from the literature to identify nodes of high, medium, and low risk in models. The exposure rate of a risk factor in the study population was *P_0_*. We generate 100,000 random *P* values of 0: 1, *P*<*P_0_* was recorded as 1 (ie, exposure), and *P*>*P_0_* was recorded as 0 (ie, nonexposure). Each risk factor simulates a column of data, summarizing the exposure of 100,000 community residents to each risk factor.

### Statistical Analysis

#### Missing Data Interpolation

Among 11,692 participants, 1 individual did not have information on hypertension, and 2556 individuals lacked information on blood lipid levels. In accordance with previous studies [12,19,20], the incomplete data for hypertension and dyslipidemia were imputed simultaneously by multiple imputations (n=25) using the R package MICE (Stef van Buuren) [21]. Based on the Akaike information criterion value, 2 of the interpolation datasets were selected and the same analysis was performed on the selected interpolation dataset to identify results that were likely to be robust. The detailed data report of the other interpolation set is presented in the Tables S2-S5 in Multimedia Appendix 1.

#### Model Evaluation

First, we assessed the discrimination for the Rothman-Keller model and the logistic regression model using the area under the receiver operating characteristic curve (AUC). Our primary objective was to determine whether the predictive capability of the Rothman-Keller model surpassed that of the conventional logistic regression model, particularly in a multiclassification situation. When the increase in AUC is not statistically significant, its interpretation can become challenging [22-24]. Therefore, in addition to the AUC, we incorporated the absolute net reclassification improvement (NRI) to evaluate the relative performance of the 2 models. If the absolute NRI is greater than 0 or less than or equal to 1, it indicates a positive improvement, showing that the predictive ability of the new index has improved compared to the old index for stroke events. Conversely, if absolute NRI is less than 0 or greater than or equal to −1, it signifies a negative change, suggesting an improvement in the predictive ability of the new model for no stroke events. If the absolute NRI is equal to 0, it means that the new model shows no improvement. In our study, we analyzed the reclassification and absolute NRI for individuals who experienced a stroke event and those who did not. For individuals who have had a stroke, being reclassified into a higher-risk group was deemed an improvement in classification, whereas being reclassified into a lower-risk group was considered a failure.

A *P* value less than .05 was considered to indicate statistical significance. The statistical analyses were performed using R 4.2 software (R Core Team).

### Ethical Considerations

The ethics review committee of The People’s Hospital of Ningxia Hui Autonomous Region approved this study (approval number: 2020-KY-053). Patients provided informed consent for using the data. Data were deidentified. No compensation was provided.

## Results

### Characteristics of the Study Cohort

**Figure 1.**
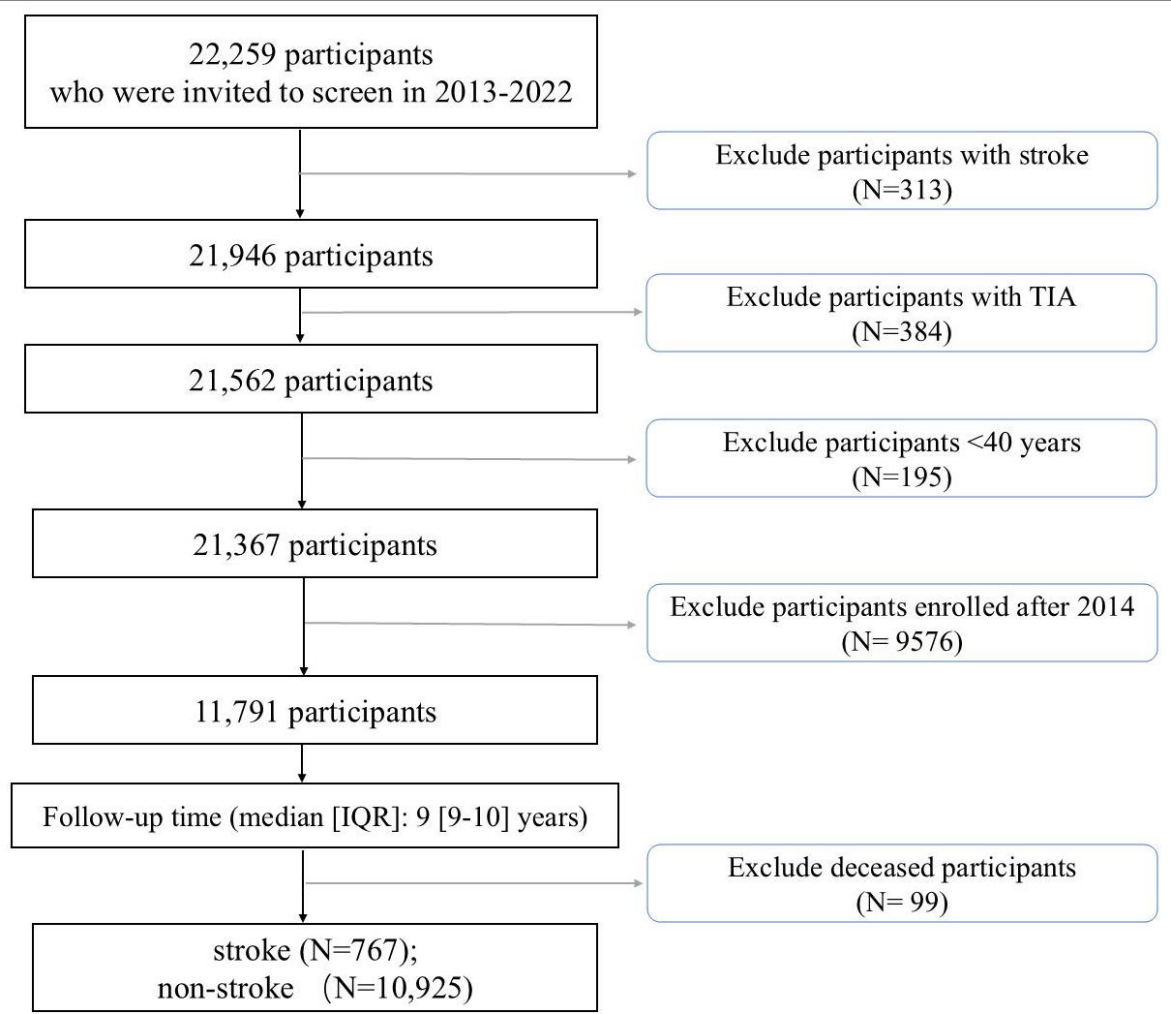
Study participants screening process.

A total of 22,259 community residents were recruited in 2013. After excluding individuals with a history of stroke (n=313), and TIA (n=384), as well as participants younger than 40 years old (n=195) and those recruited after 2014 (n=9576), 11,791 participants were included in the follow-up cohort. After a 10-year follow-up period, 99 participants were lost to follow-up. Finally, a total of 11,692 eligible participants were included in the final analysis (Figure 1). A total of 767 participants (6.6%) had a stroke by the end of the follow-up period. Based on the “8+2” stroke risk score, the 10-year stroke incidence among the 3 stroke risk groups of the community residents was as follows: low-risk group 4.47% (n=8908, 398 stroke cases); medium-risk group 14.71% (n=1074, 158 stroke cases); and high-risk group 12.34% (n=1710, 211 stroke cases) (Table 1). Kaplan-Meier survival curves are shown in Figure S1 in Multimedia Appendix 1.

**Table 1.** Characteristics of participants based on different risk levels according to the “8+2” stroke risk score at baseline in Ningxia (N=11,692).

Characteristics	All participants, N (%)	High-risk participants, n (%)	Medium-risk participants, n (%)	Low-risk participants, n (%)
Total	11,692 (100)	1710 (14.6)	1074 (9.2)	8908 (76.2)
Age (years)				
40‐49	5457 (46.7)	468 (27.4)	227 (21.1)	4762 (53.5)
50‐59	3298 (28.2)	600 (35.1)	318 (29.6)	2380 (26.7)
60‐69	2050 (17.5)	500 (29.2)	336 (31.3)	1214 (13.6)
≥70	887 (7.6)	142 (8.3)	193 (18.0)	552 (6.2)
Sex				
Female	5797 (49.6)	919 (53.7)	608 (56.6)	4270 (47.9)
Male	5895 (50.4)	791 (46.3)	466 (43.4)	4638 (52.1)
District				
Urban	6057 (51.8)	1212 (70.9)	189 (17.6)	4656 (52.3)
Rural	5635 (48.2)	498 (29.1)	885 (82.4)	4252 (47.7)
Family history of stroke				
Yes	361 (3.1)	289 (16.9)	15 (1.4)	57 (0.6)
No	11,331 (96.9)	1421 (83.1)	1059 (98.6)	8851 (99.4)
Heart disease				
Yes	461 (3.9)	265 (15.5)	196 (18.2)	0 (0)
No	11,231 (96.1)	1445 (84.5)	878 (81.8)	8908 (100)
Hypertension				
Yes	1684 (14.4)	903 (52.8)	781 (72.7)	0 (0)
No	10,008 (85.6)	807 (47.2)	293 (27.3)	8908 (100)
Dyslipidemia				
Yes	1639 (14.0)	1343 (78.5)	113 (10.5)	183 (2.1)
No	10,053 (86.0)	367 (21.5)	961 (89.5)	8725 (97.9)
Diabetes				
Yes	395 (3.4)	190 (11.1)	205 (19.1)	0 (0)
No	11,297 (96.6)	1520 (88.9)	869 (80.9)	8908 (100)
Smoking				
Yes	1355 (11.6)	439 (25.7)	92 (8.6)	824 (9.3)
No	10,337 (88.4)	1271 (74.3)	982 (91.4)	8084 (90.7)
Overweight				
Yes	2362 (20.2)	1100 (64.3)	267 (24.9)	995 (11.2)
No	9330 (79.8)	610 (35.7)	807 (75.1)	7913 (88.8)
Physical inactivity				
Yes	2983 (25.5)	1204 (70.4)	198 (18.4)	1581 (17.7)
No	8709 (74.5)	506 (29.6)	876 (81.6)	7327 (82.3)

### Model Construction

The baseline incidence ratio (*ρ*) of 8 factors was obtained to calculate the population attributable risk percentage (PAR%) through a previous study [18]. The OR values of these factors were assessed using a logistic regression model. The parameters of the logistic model and the Rothman-Keller model are shown in Table 2.

**Table 2.** Parameters of risk exposure factors in the logistic model and Rothman-Keller model.

Risk factor	P_i_ ^a^	OR*_i_*^b^ (95% CI)	β_*i*_^c^	PAR (%)^d^	ρ^e^	S^f^
Hypertension						
Yes	0.580	3.00 (2.49‐3.59)	1.097	50.5	0.495	1.485
No	0.420	1			0.495	0.495
Diabetes						
Yes	0.297	2.21 (1.67‐2.90)	.793	17.4	0.826	1.825
No	0.703	1			0.826	0.826
Dyslipidemia						
Yes	0.297	0.94 (0.76‐1.17)	–.060	19.6	0.804	0.756
No	0.703	1			0.804	0.804
Heart diseases						
Yes	0.691	1.24 (0.89‐1.69)	.211	50.4	0.496	0.615
No	0.309	1			0.496	0.496
Smoking						
Yes	0.213	0.88 (0.69‐1.10)	–.133	8.2	0.918	0.808
No	0.787	1			0.918	0.918
Overweight						
Yes	0.054	1.51 (1.27‐1.79)	.411	2.1	0.979	1.478
No	0.946	1			0.979	0.979
Physical inactivity						
Yes	0.515	1.01 (0.85‐1.20)	.010	34.9	0.651	0.658
No	0.485	1			0.651	0.651
Family history of stroke						
Yes	0.085	1.40 (1.01‐1.92)	.338	5.1	0.949	1.329
No	0.915	1			0.949	0.949

a P_i_: the exposure rate of individuals exposed to a risk factor in the whole population

b OR_i_: the odds ratios of exposure to a risk factor;

c β_i_ is the beta coefficient

d PAR%: population attributed risk percentage

e ρ*:* baseline morbidity ratio

f S*:* risk score

After ranking the stroke incidence risk based on a dataset of 100,000 random entries, 2 nodes were selected for subdividing the risk groups into low-risk, medium-risk, and high-risk categories using the logistic regression model: node a (ID=25844, risk prediction score=0.06168760) and node b (ID=77778, risk prediction score=0.16451650). In addition, for the Rothman-Keller model, 2 other nodes were chosen for the same purpose: node A (ID=25426, risk prediction score=0.0021993391) and node B (ID=64553, risk prediction score =0.0047898060) (Figure S2 in Multimedia Appendix 1).

### Evaluation of the Model

The sensitivity, specificity, and AUC of the “8+2” stroke risk score, the Rothman-Keller model, and the logistic regression model are presented in Table 3. The logistic regression model and the Rothman-Keller model demonstrated significant differences in AUC values compared to the “8+2” stroke risk score (Z=2.60, *P*<.05; Z=3.47, *P*<.05, respectively). However, no difference was observed between the logistic regression model and the Rothman-Keller model (Z=0.688, *P*>.05). The comparison of receiver operating characteristic curve is shown in Figure S3 in Multimedia Appendix 1.

**Table 3.** The discrimination of “8+2” stroke risk score, logistic and Rothman-Keller model.

	Sensitivity	Specificity	AUC^a^ (95% CI)	Z value (*P*) ^b^	Z value (*P*)^c^
“8+2” stroke risk score	0.48	0.79	0.627 (0.619‐0.636)		
Logistic model	0.41	0.85	0.649 (0.641‐0.658)	3.47 (*P*=.001)	
Rothman-Keller model	0.52	0.74	0.646 (0.637‐0.654)	2.60 (*P*=.009)	0.688 (*P*=.492)

a AUC: area under the curve

b :Versus “8+2” stroke risk score;

c :Versus logistic model

From the NRI, we found that the majority of participants remained at the same level of risk for developing a stroke as predicted by the “8+2” stroke risk score, the logistic regression model, and the Rothman-Keller model (ie, along the diagonal from the lower left to the upper right). However, some community residents were reclassified as having a different level of risk for developing stroke (Figure S4 in Multimedia Appendix 1). According to the “8+2” stroke risk score, the NRI for reclassification of stroke events by the Rothman-Keller model was 7.8%, while the NRI for nonstroke events was −2.7%. The absolute NRI was then estimated to be 0.051 (*P*=.01), calculated using the sum of the net estimated for individuals who developed a stroke and those who did not. The NRI for the logistic regression model was 1.7% for stroke events and −0.7% for nonstroke events, resulting in an absolute NRI of 0.010 (*P*=.62) .

## Discussion

### Principal Findings

This cohort study followed 11,692 individuals aged 40 years and older for a duration of 10 years. According to the “8+2” stroke risk score, the stroke incidence in the low-risk (n=8908), medium-risk (n=1074), and high-risk groups (n=1710) was 4.5%, 14.7%, and 12.3%, respectively. We developed a logistic regression model and a Rothman-Keller model to validate and optimize the risk score. Through a comparative analysis of the performance of the 3 models, we found that the Rothman-Keller model exhibited the best performance.

We verified the efficacy of the model using an actual database. There was no significant difference in the AUC values between the logistic regression model and the Rothman-Keller model. To evaluate model performance more accurately, we used the NRI for a more in-depth analysis. The NRI assesses the effects of low-, medium-, and high-risk reclassification for both stroke and nonstroke events, resulting in a net reclassification that provides a more accurate estimate than that obtained with other approaches [23]. Positive values of the stroke NRI indicate that the model effectively identifies patients with stroke, enabling physicians to initiate targeted detection or treatment to prevent stroke events. In contrast, a decrease in the NRI for nonstroke events suggests that community residents with a low or medium risk as determined by the “8+2” stroke risk score may actually be at a higher risk of having a stroke. Based on the overall NRI, we conclude that the Rothman-Keller model enhances the reclassification of both stroke and nonstroke events [25]. This finding aligns with the results of previous studies. Researchers used the Rothman-Keller model to predict the likelihood of mild cognitive impairment in older Chinese individuals. Upon validation with actual population data, it was found that the model had appropriate accuracy and performed well in terms of predictive efficacy [16,26]. The model can be adjusted and optimized based on new research data and epidemiological changes, thereby maintaining its predictive power in a timely manner. Its methodology can be applied to risk assessments for other populations and chronic diseases, demonstrating significant universality.

There are many model studies aimed at predicting stroke risk [27-30]. For instance, SCORE2 is a risk assessment tool developed using extensive data from a large number of European populations. It is designed to evaluate the risk of cardiovascular disease in both men and women across 4 distinct risk areas in Europe within a 10-year period [31]. While this tool is widely applied, it has limitations in terms of ethnicity and geography. Significant prediction errors may occur when applying it to populations with considerable differences [9,32]. A study estimated the 10-year risk of stroke based on a cohort analysis and found that factors such as age, systolic blood pressure, diastolic blood pressure, FHS, atrial fibrillation, diabetes, and others can significantly predict the incidence of stroke [27]. Compared with other models, this study developed a Rothman-Keller model based on questionnaire information to identify new risk nodes through simulation datasets, which provided a basis for stroke prevention. Furthermore, the model’s predictive power and accuracy were verified using real-world data. Logistic regression analysis indicated that smoking, heart disease, dyslipidemia, and physical inactivity were not related to stroke, which may be attributed to variations in demographic and stroke subtypes differences. This result was consistent with the results of previous Mendelian randomization studies [33-37].

The limitations of this study are as follows: first, the dataset used for verification only included participants from only one region. Studies in other provinces are necessary to evaluate the efficacy of our model. When research results are extrapolated to other populations with significant differences, it may be essential to consider the exposure rates of risk factors and OR or RR values for those populations in order to update and optimize the model. This process enhances the predictive accuracy and applicability of the Rothman-Keller model. Second, we were unable to perform a subgroup analysis on the various types of stroke. The primary objective of the program is to identify and intervene with high-risk populations to prevent the occurrence of stroke or reduce its risk, rather than focusing on the subtypes of ischemic stroke and hemorrhagic stroke. Furthermore, given the limitations of medical resources and the acceptability of screening, the program may prioritize the implementation of more accessible and universal preventive measures. These measures include controlling blood pressure, quitting smoking, and increasing physical activity, all of which are effective in preventing both ischemic stroke and hemorrhagic stroke [38,39]. Third, during the decade, participants may have received lifestyle interventions, pharmaceutical treatments, and early clinical treatment that influenced the incidence of stroke events. However, in the medium- and high-risk groups for stroke, these patients represent a certain proportion. Our model primarily assesses the variations in the initial screening judgments, and the outcome events remain consistent across different models, making it unlikely to influence the study results. Finally, the low sensitivity observed in this study may be attributed to the lack of several important stroke prediction factors from the risk scoring scale, thereby limiting its predictive ability. In 2021, the Guidelines for Stroke Prevention and Treatment in China recommended including homocysteine testing in routine screening and conducting carotid artery examinations for high-risk populations when conditions permit. Our research findings further support this recommendation. Although this study has some limitations, it also presents several advantages. The diagnostic criteria for risk factors in this project are based on relevant guidelines and standards established by the China Health Commission. Staff members undergo standardized training and assessment, and only those who pass the assessment are qualified to conduct screening tasks. Therefore, the identification of risk factors in this study had high accuracy and credibility. We used the binomial distribution of risk factors to construct a random dataset of 100,000 community residents, which allowed us to determine the high-, medium-, and low-risk boundary values of the models. The variables in the model were easily obtained and predicted estimates could be derived through straightforward calculations. We included a substantial number of community residences from the CSHPSIP over a decade for external validation, which exhibited good discrimination and calibration.

### Conclusions

In conclusion, the Rothman-Keller model may improve the predictive efficacy of stroke screening models. In the future, verification will need to be carried out in a wider population and combined with more risk factors. The Rothman-Keller model for assessing individualized stroke risk, combined with interactive information platforms for health education, is beneficial for decreasing the incidence of stroke among high-risk groups.

## Supplementary material

10.2196/72497 Multimedia Appendix 1 Supplementary data to this article can be found in Multimedia Appendix 1.

## Abbreviations

AUC: area under the receiver operating characteristic curve
CNSSS: China National Stroke Screening Survey
CSHPSIP: China Stroke High-risk Population Screening and Intervention Program
CSPPC: China Stroke Prevention Project Committee
FHS: family history of stroke
GBD: Global Burden of Disease
*ICD-10*: *International Statistical Classification of Diseases and Related Health Problems 10th Revision*
NRI: net reclassification improvement
TIA: transient ischemic attack
TRIPOD: Transparent reporting of multivariable prediction model for Individual Prognosis or Diagnosis
