# Supplementary material for: Validation and Refinement of Scores to Predict Stroke Risk: Prospective Cohort Study
Source: JMIR Public Health Surveill. 2025 Aug 21;11:e72497. doi: 10.2196/72497 (PMC12395389; doi:10.2196/72497)
Supplement: Multimedia Appendix 1 [file publichealth-v11-e72497-s001.docx]

**Data quality control**

The Community health service center staff mobilize the target population to identify the subjects for screening, create a roster, notify the subjects of the time and location for the on-site examination, verify their identity information, obtain signed informed consent forms. Eligible community residents were administered a questionnaire and a physical examination. The physical examination includes items such as height, weight, waist circumference, and blood pressure for the subjects of the project. Trained professionals use cardiac auscultation to determine whether the project subjects have regular heart rhythms. If an irregular heartbeat is detected, an electrocardiogram (ECG) examination is required to clarify whether it is atrial fibrillation. The history of stroke and TIA among community residents is first self-reported and then assessed by a neurologist. The neurologist provides the definitive diagnosis based on the medical record sheet from a secondary or tertiary hospital or supported by a CT scan report.

To ensure the smooth implementation and quality control of the project, a quality control system is established. Training is conducted through a tiered approach for screening techniques and personnel, which is integrated into every aspect of the project's implementation. Before the survey, uniform training is provided to the community health service center staff, including qualified physicians and nurses and public health personnel, and only those who pass the assessment are qualified to participate in the project's work. During the survey, standardized questionnaires, technical specifications, and diagnostic criteria developed by the National Health Commission's Stroke Project Committee are used. The survey is conducted face-to-face and supervised by higher-level institutions. After the survey, the staff at the project screening sites review the data, which is then reported through the stroke screening platform information system to the "China Stroke Data Center" for national data collection, review, and cleaning.

**
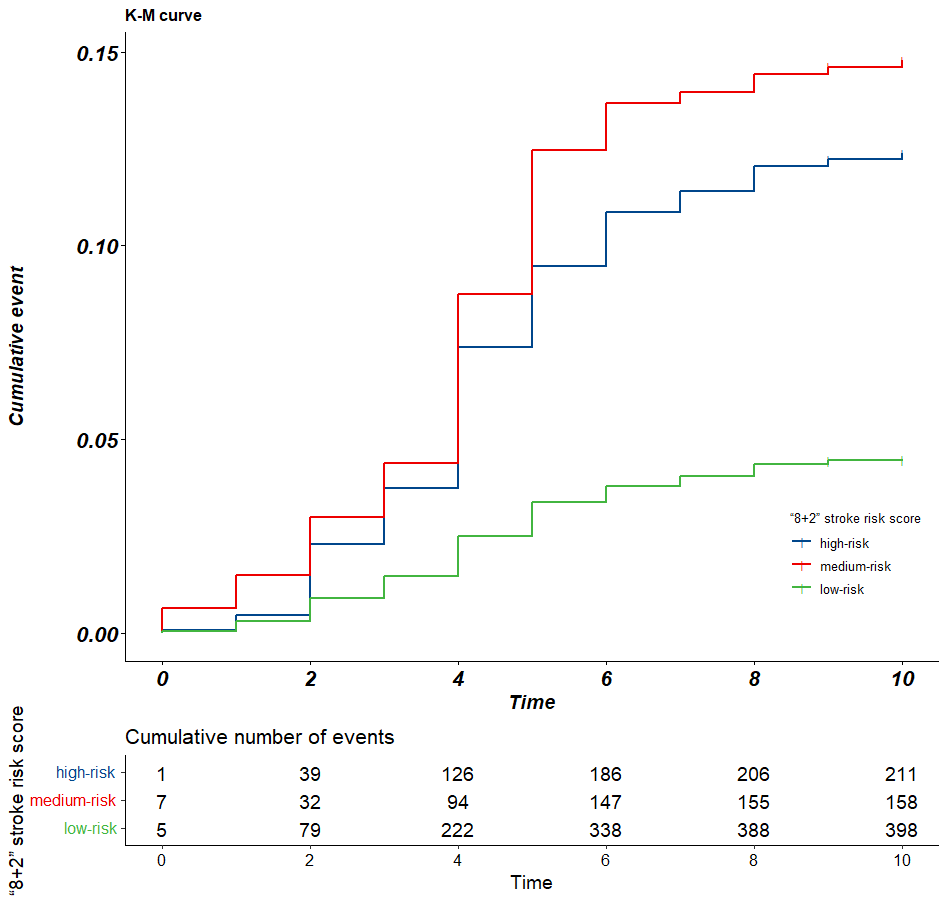
**

**Figure S1.** 10-year incidence of stroke in the “8+2” questionnaire at high-, medium- and low-risk groups (Time /year)


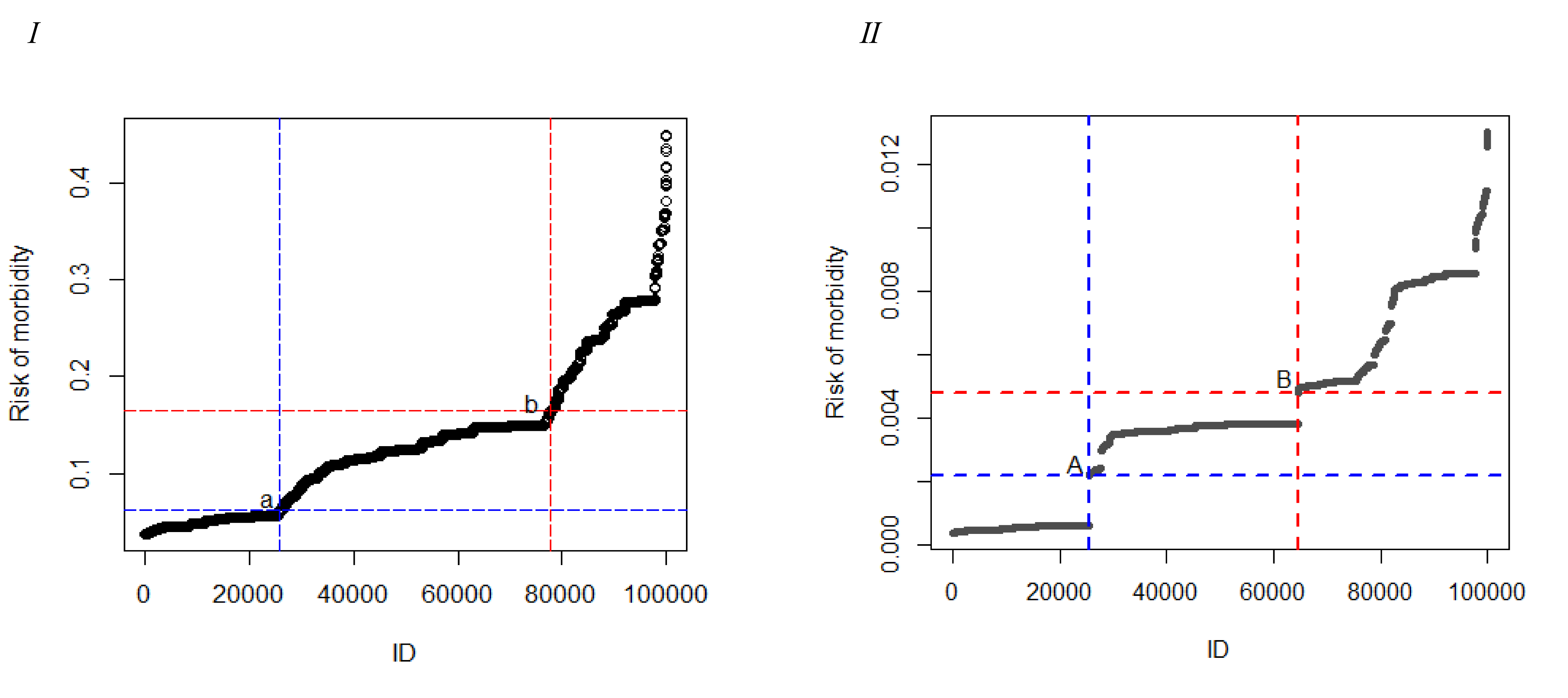


**Figure S2.** The predictive analysis of models (I: Logistic model; Ⅱ: Rothman-Keller model)**：**The horizontal axis is the ID of measured individuals, and the vertical axis (risk of morbidity) represents the individual's relative risk level of stroke compared to the overall population, which is a relative value without units. A/a: The cut-off points between low- and medium-risk; B/b: The cut-off points between medium- and high-risk; Red and blue dotted lines: Guides for the demarcation point. The different scales of the proportions are due to the varying overall ranges of risk values calculated by the logistic model and the Rothman-Keller model.


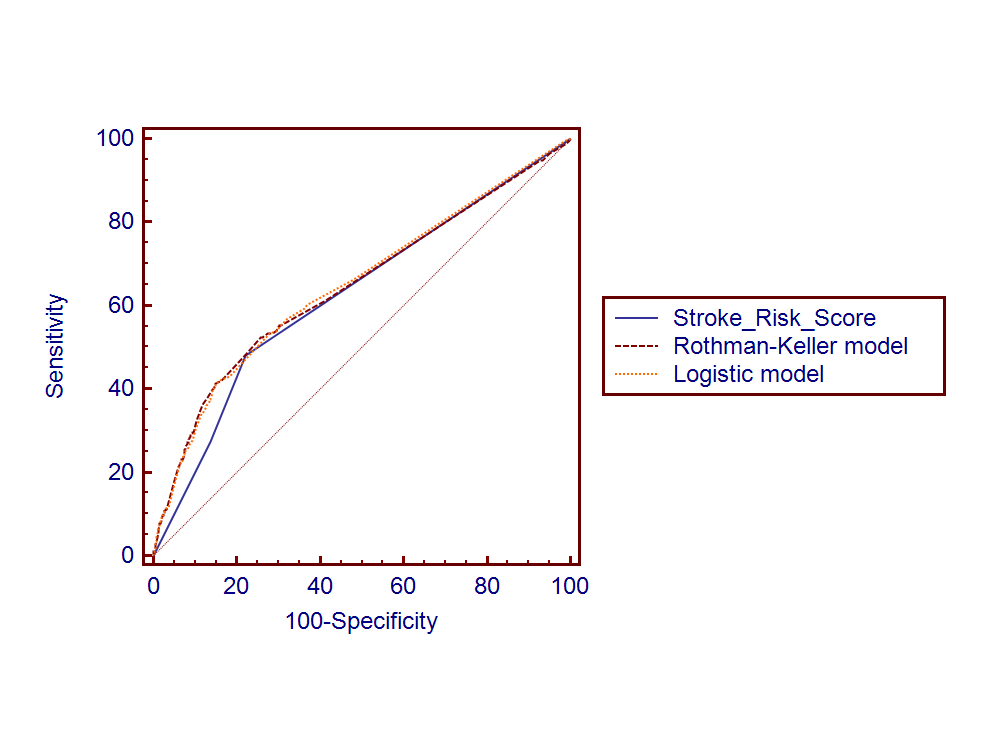


**Figure S3.** The receiver operating characteristic curve of models

Supplement Figure 4. The number of participants in different categories of stroke risk with reclassification

**Table S1.** Classification criteria for the “8+2” stroke risk assessment

| Risk factors | | Judging criteria |
| --- | --- | --- |
| Hypertension | | Any of the following is sufficient:  a) History of hypertension (diagnosed by a secondary hospital or above).  b) The results of this screening measurement indicate an increase in blood pressure (systolic ≥140mmHg or diastolic ≥90mmHg). |
| Dyslipidemia | | Any of the following is sufficient:  a) Previous medical history (diagnosed by a secondary hospital or above).  b) In this field measurement, total cholesterol ≥6.22 mmol/L (240 mg/dl), Triglyceride ≥2.3mmol/L (200 mg/dl), high-density lipoprotein <1.04mmol/L (40 mg/dl), low density lipoprotein ≥4.1 mmol/L(160 mg/dl), one or more of which can be identified as dyslipidemia. |
| Diabetes | | Any of the following is sufficient:  a) Previous medical history (diagnosed by a secondary hospital or above).  b) The on-site measurement showed elevated blood glucose (random blood glucose ≥11.0 mmol/L or fasting blood glucose ≥7.0mmol/L). |
| Smoking | | Active smokers: Those who have smoked continuously or cumulatively for 6 months or more in their lifetime.  Quitters: Smokers were no longer smokers at the time of the survey and had been for more than 6 months. |
| Atrial fibrillation or valvular heart disease | | Any of the following is sufficient:   1. Previous medical history (diagnosed by a secondary hospital or above). 2. This ECG shows atrial fibrillation. |
| Overweight | | BMI≥26 For overweight. [(BMI= weight (kg)/$\mathrm{height}^{2}$ ($m^{2}$)] |
| Little physical exercise | | Exercise ≥3 times a week, each moderate intensity or more exercise ≥30 minutes, or engaged in moderate or heavy manual labor is considered as regular physical exercise. Otherwise, lack of exercise. |
| Family history of stroke | | A definite diagnosis was made in a secondary or higher hospital.  a）History of previous stroke. A definite diagnosis in a secondary or higher hospital; The CT report.  b）TIA history. A definite diagnosis in a secondary or higher hospital; The CT report. |
| Evaluation result | High risk | Three or more of these risk factors are present |
|  |  | Previous history of stroke |
|  |  | History of transient ischemic attacks (TIA) |
|  | Moderate risk | People with less than 3 risk factors and one of hypertension、diabetes or atrial fibrillation |
|  | Low risk | Have less than 3 risk factors and no chronic diseases |

**Table S2.** Distribution of people at different risk levels as determined by the “8+2” Stroke Risk Score (N=11692) [The other interpolation data set]

| Characteristics | | All | | High-risk | | Medium-risk | | Low-risk | |
| --- | --- | --- | --- | --- | --- | --- | --- | --- | --- |
|  |  | N | % | N | % | N | % | N | % |
| Total |  | 11692 | 100 | 1710 | 14.6 | 1074 | 9.2 | 8908 | 76.2 |
| Age | 40-49 | 5457 | 46.7 | 468 | 27.4 | 227 | 21.1 | 4762 | 53.5 |
|  | 50-59 | 3298 | 28.2 | 600 | 35.1 | 318 | 29.6 | 2380 | 26.7 |
|  | 60-69 | 2050 | 17.5 | 500 | 29.2 | 336 | 31.3 | 1214 | 13.6 |
|  | ≥70 | 887 | 7.6 | 142 | 8.3 | 193 | 18.0 | 552 | 6.2 |
| Gender | female | 5797 | 49.6 | 919 | 53.7 | 608 | 56.6 | 4270 | 47.9 |
|  | male | 5895 | 50.4 | 791 | 46.3 | 466 | 43.4 | 4638 | 52.1 |
| District | country | 6057 | 51.8 | 1212 | 70.9 | 189 | 17.6 | 4656 | 52.3 |
|  | village | 5635 | 48.2 | 498 | 29.1 | 885 | 82.4 | 4252 | 47.7 |
| Family history of stroke | no | 11331 | 96.9 | 1421 | 83.1 | 1059 | 98.6 | 8851 | 99.4 |
|  | yes | 361 | 3.1 | 289 | 16.9 | 15 | 1.4 | 57 | 0.6 |
| Heart disease | no | 11231 | 96.1 | 1445 | 84.5 | 878 | 81.8 | 8908 | 100.0 |
|  | yes | 461 | 3.9 | 265 | 15.5 | 196 | 18.2 | 0 | 0.0 |
| Hypertension | no | 10008 | 85.6 | 807 | 47.2 | 293 | 27.3 | 8908 | 100.0 |
|  | yes | 1684 | 14.4 | 903 | 52.8 | 781 | 72.7 | 0 | 0.0 |
| Dyslipidemia | no | 10076 | 86.2 | 386 | 22.6 | 964 | 89.8 | 8726 | 98.0 |
|  | yes | 1616 | 13.8 | 1324 | 77.4 | 110 | 10.2 | 182 | 2.0 |
| Diabetes | no | 11297 | 96.6 | 1520 | 88.9 | 869 | 80.9 | 8908 | 100.0 |
|  | yes | 395 | 3.4 | 190 | 11.1 | 205 | 19.1 | 0 | 0.0 |
| Smoking | no | 10337 | 88.4 | 1271 | 74.3 | 982 | 91.4 | 8084 | 90.7 |
|  | yes | 1355 | 11.6 | 439 | 25.7 | 92 | 8.6 | 824 | 9.3 |
| Overweight | no | 9330 | 79.8 | 610 | 35.7 | 807 | 75.1 | 7913 | 88.8 |
|  | yes | 2362 | 20.2 | 1100 | 64.3 | 267 | 24.9 | 995 | 11.2 |
| Physical inactivity | no | 8709 | 74.5 | 506 | 29.6 | 876 | 81.6 | 7327 | 82.3 |
|  | yes | 2983 | 25.5 | 1204 | 70.4 | 198 | 18.4 | 1581 | 17.7 |

**Table S3.** Parameters of risk exposure factors in the Logistic and Rothman-Keller model [The other interpolation data set]

| Risk factor | *P_i_* | *OR_i_* (95% confidence intervals) | β*_i_* | *PAR*（%） | *ρ* | *S* |
| --- | --- | --- | --- | --- | --- | --- |
| Hypertension |  |  |  |  |  |  |
| yes | 0.580 | 3.01 (2.51-3.61) | 1.103 | 50.5 | 0.495 | 1.490 |
| no | 0.420 | 1 |  |  | 0.495 | 0.495 |
| Diabetes |  |  |  |  |  |  |
| yes | 0.297 | 2.22 (1.67-2.91) | 0.796 | 17.4 | 0.826 | 1.834 |
| no | 0.703 | 1 |  |  | 0.826 | 0.826 |
| Dyslipidemia |  |  |  |  |  |  |
| yes | 0.297 | 0.92 (0.74-1.14) | -0.086 | 19.6 | 0.804 | 0.740 |
| no | 0.703 | 1 |  |  | 0.804 | 0.804 |
| Heart diseases |  |  |  |  |  |  |
| yes | 0.691 | 1.23 (0.89-1.68) | 0.211 | 50.4 | 0.496 | 0.610 |
| no | 0.309 | 1 |  |  | 0.496 | 0.496 |
| Smoking |  |  |  |  |  |  |
| yes | 0.213 | 0.88 (0.69-1.10) | -0.130 | 8.2 | 0.918 | 0.808 |
| no | 0.787 | 1 |  |  | 0.918 | 0.918 |
| Overweight |  |  |  |  |  |  |
| yes | 0.054 | 1.51 (1.27-1.80) | 0.415 | 2.1 | 0.979 | 1.478 |
| no | 0.946 | 1 |  |  | 0.979 | 0.979 |
| Physical inactivity |  |  |  |  |  |  |
| yes | 0.515 | 1.02 (0.85-1.21) | 0.016 | 34.9 | 0.651 | 0.664 |
| no | 0.485 | 1 |  |  | 0.651 | 0.651 |
| Family history of stroke |  |  |  |  |  |  |
| yes | 0.085 | 1.42 (1.02-1.94) | 0.348 | 5.1 | 0.949 | 1.348 |
| no | 0.915 | 1 |  |  | 0.949 | 0.949 |

Abbreviation: P_i_: the exposure rate of individuals exposed to a risk factor in the whole population; OR_i_: the odd ratios of exposure to a risk factor; CI: confidence intervals; β_i_ is the beta coefficients; PAR%: population attributed risk percentage; ***ρ:***  baseline morbidity ratio; ***S:*** risk score.

**Table S4.** The receiver operating characteristic curve of models in different models with reclassification [The other interpolation data set]

|  | Sensitivity | Specificity | AUC (95% CI) | Z value (P) ^a^ | Z value (P)^b^ |
| --- | --- | --- | --- | --- | --- |
| “8+2” Stroke Risk Score | 0.48 | 0.79 | 0.627(0.619-0.636) |  |  |
| Logistic model | 0.41 | 0.85 | 0.646(0.637-0.654) | 2.61 (P<0.05) |  |
| Rothman-Keller model | 0.52 | 0.74 | 0.646(0.637-0.654) | 2.67 (P<0.005) | 0.795 (P>0.05) |

Abbreviation: AUC:area under the curve; CI:confidence intervals;

^a^：Vs “8+2” Stroke Risk Score; **^b^:** Vs Logistic model

**Table S5.** The net reclassification indices in different models with reclassification [The other interpolation data set]

| Net reclassification indices | Stroke events | Non-stroke events | Absolute (P) |
| --- | --- | --- | --- |
| “8+2” Stroke Risk Score vs Rothman-Keller model | 0.078 | -0.027 | 0.051 (P<0.05) |
| “8+2” Stroke Risk Score vs Logistic model | -0.030 | 0.026 | -0.004 (P>0.05) |
